# Structural and Spectroscopic Evidence of Hydrogen Bonded Anionic Wires in Protic Ionic Liquids

**DOI:** 10.1002/cphc.70581

**Published:** 2026-09-29

**Authors:** Maham Azhar, Lasse Hunger, Loai Al‐Sheakh, Andreas Appelhagen, Lennart Kruse, Ralf Ludwig

**Affiliations:** ^1^ Institut für Chemie Abteilung Physikalische Chemie Universität Rostock Rostock Germany; ^2^ Department Life, Light & Matter Universität Rostock Rostock Germany; ^3^ Leibniz Institut für Katalyse an der Universität Rostock e.V. Rostock Germany

**Keywords:** hydrogen bonds, infrared spectroscopy, nuclear magnetic resonance spectroscopy, quantum chemistry, X‐ray crystallography

## Abstract

Hydrogen Bonding (H‐bonds) between ions of like charge challenges the fundamental principle of electrostatics that opposite charges attract and like charges repel. This study provides direct structural and spectroscopic evidence of H‐bonded anionic wires in protic ionic liquids (PILs), which consist of triethylammonium cations and hydrogen dicarboxylate anions. The anionic wires were identified by X‐ray crystallography in the solid state, wherein the anions are linked by ^−^OH···O^−^ type H‐bonds. The H‐bonds within the anionic wire are significantly stronger than those between cation and anion despite repulsive Coulomb interaction in the first and attractive ones in the latter case. Analysis by nuclear magnetic resonance (NMR) and infrared (IR) spectroscopy suggests that the binding motifs of the solid structure are mainly preserved in the liquid state of the supercooled PILs. This conclusion is drawn from density functional theory (DFT) calculations of the same structural motifs as observed in the solid state. The calculated ^1^H NMR chemical shifts and IR C═O frequencies for these H‐bond structures correspond well with the measured spectroscopic signatures. The anionic chains remain intact even when gradually removing the counterions. Our findings highlight the fundamental role of hydrogen bonding for the unusual attractive interaction between ions of like charge.

## Graphical abstract

Anionic wires of deprotonated carboxylic acids challenge the general wisdom that like charges repel. The hydrogen bond motif observed in the X‐ray structure is even preserved in the liquid state of the material as shown by *δ* ^1^H NMR spectroscopy. The experimental and theoretical findings provide deep insight into the nature of hydrogen bonding. The highly delocalized protons in the wires hold promise for materials with excellent proton conductivity.

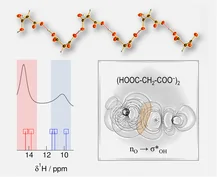

## 1 Introduction

Hydrogen bonds (H‐bonds) are ubiquitous in nature and essential for life [1]. In liquid water, a flickering three‐dimensional network of H‐bonds is responsible for its unique properties [2, 3]. In proteins, H‐bonds stabilize helices and beta sheets and control the fold into functional shapes [4, 5]. The base pairs in double‐stranded DNA are held together by double and triple H‐bonds, allowing them to unzip for replication without falling apart [6]. H‐bonds play an important role even in ionic liquids (ILs), although they are an order of magnitude weaker than the ionic interactions between cations and anions in this liquid material [7, 8, 9, 10]. This may not be surprising for H‐bonds between ions of opposite charges, which further strengthen the attractive interaction between cation and anion. In contrast, H‐bonds between ions of like charge seem to be unusual, challenging the general wisdom that opposite charges attract but like charges repel. Nevertheless, H‐bonds between cations have been observed in hydroxy‐functionalized ILs [11, 12]. In linear and cyclic clusters, the interaction between ions of like charge is additionally strengthened by cooperative H‐bonds. The recipe for enhanced formation of cationic clusters includes weakly interacting anions, polarizable cations, and longer alkyl chains, which increases the distance between the hydroxy functional groups and the positively charged ring system of the cations, thereby reducing Coulomb repulsion [13, 14]. More recently, we replaced the hydroxy‐ by carboxy‐functionalized cations in similar ILs. We observed doubly H‐bonded cationic dimers in the solid, liquid, and gas phases [15, 16, 17]. The carboxy‐functionalized ILs exhibit the same structural motif as that known for formic and acetic acid in the gas phase [18, 19]. Obviously, the double H‐bonds are strong enough to overcome the repulsive Coulomb forces between the cations in all phases. If H‐bonds are strong and directional, microscopic systems display the H‐bond motifs present in the bulk.

In this work, we take on the challenge of finding similar structural motifs for anionic clusters in protic ionic liquids (PILs). However, the synthesis of PILs comprising anionic dimers or even larger like‐charged clusters is much more difficult to realize and thus rarely discussed in the literature [20, 21, 22]. Herein, we show that the syntheses from triethylamine (TEA) and dicarboxylic acids lead to PILs exhibiting such anionic structural motifs. This kind of PILs have been synthesized because herein both ions provide single donor functional groups NH and OH, which allow studying the competition between well‐defined cation–anion ^+^NH···O^−^ and anion–anion ^−^OH···O^−^ types of H‐bonds. Here, fundamental understanding of interionic interaction, in particular the nature of inter anionic interaction and the important role of H‐bonds, is required. Instead of doubly H‐bonded anions, we observed infinite chains of singly H‐bonded anions. The H‐bonded anionic wires are flanked by additional H‐bonds between cation and anion. X‐ray crystallography provide evidence that the anion–anion H‐bonds are stronger than the cation‐anion H‐bonds despite repulsive Coulomb interaction in the first and attractive ones in the latter case. The structural motifs observed in the solid state survive the transition to the liquid phase, as demonstrated by ^1^H nuclear magnetic resonance (NMR) and infrared (IR) spectroscopy. Density functional theory (DFT) calculations on differently charged cluster species clarify the role of H‐bonding between like‐charged ions and whether they can be regarded as “antielectrostatic” H‐bonds (AEHB) or rather as simple interanionic H‐bonds (IAHB) [23, 24, 25, 26, 27, 28, 29].

## 2 Results and Discussion

The starting point of our investigation has been the synthesis of the ILs or low melting salts via a simple acid–base reaction of dicarboxylic acids and TEA as the base. For studying the effect of adding one methylene group CH_2_ between the two carboxylic groups, we used two different alkanedicarboxylic acids (HOOC(CH_2_)_*n*_COOH) with *n* = 0,1, namely, ethanedionic (oxalic) and propanedionic (malonic) acid (Scheme 1) [30, 31]. The general synthesis procedure is described in the Supporting Information.

**SCHEME 1:**
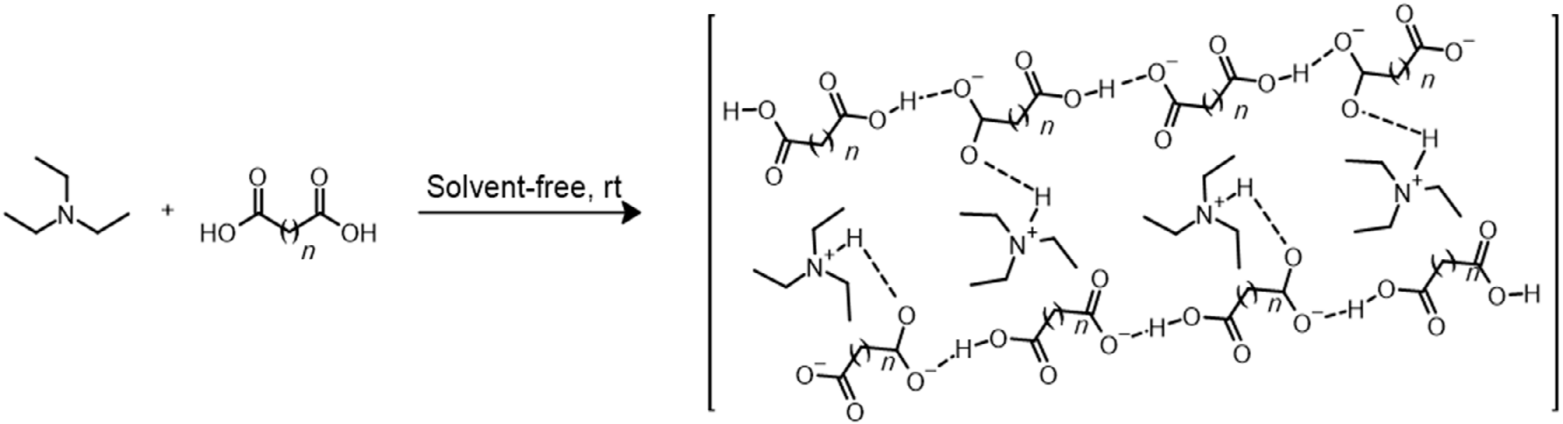
Reaction scheme for the synthesis of the protic ionic liquids of type triethylammonium hydrogen dicarboxylate [TEA][OOC(CH_2_)_*n*_COOH] with *n* = 0,1 obtained from triethyl amine and oxalic acid (*n* = 0) and malonic acid (*n* = 1), respectively.

### 2.1 Crystal Structures Exhibit Hydrogen Bonded Anionic Wires

The crystal structures of triethylammonium hydrogen oxalate and triethylammonium hydrogen malonate have been determined by X‐ray crystallography [32, 33, 34, 35]. The CDS deposition numbers are 2386822 and 2386827, respectively. Figure 1 shows the X‐ray structure of triethylammonium hydrogen oxalate. We observe hydrogen‐bonded wires consisting of alternating dicarboxylic acid molecules and doubly negatively charged oxalate anions. Thereby, the two OH groups of the molecule serve as H‐bond donors to the neighboring oxygen acceptors of the oxalate anions. The OH···O─ H‐bond between molecule and anion is very strong, suggested by the O···O distances of 2.479 Å and the elongated OH‐bond length of about 1.081 Å (see Table 1 for all geometry values). The H‐bond angle ∠(O,H,O) is 179.4°, thus perfectly linear. The triethylammonium cation exclusively forms H‐bonds to the other oxygen of the oxalate anion. The N···O distance is 2.900 Å and the NH···O distance is 1.877 Å. The NH‐bond is only 0.96 Å long, and the H‐bond angle ∠(N,H,O) is 160.5°, both suggesting moderate H‐bonds despite the Coulomb attraction between the opposite charged ions. Because we were aiming for singly H‐bonded anionic wires, we switched to triethylammonium malonate synthesized from malonic acid, which has a significantly larger pK_a1_ value than oxalic acid (pK_a1_ = 2.83 vs. pK_a1_ = 1.23) [36]. The oxalate and malonate anions only differ by introducing a single methylene group between the charged entities.

**FIGURE 1:**
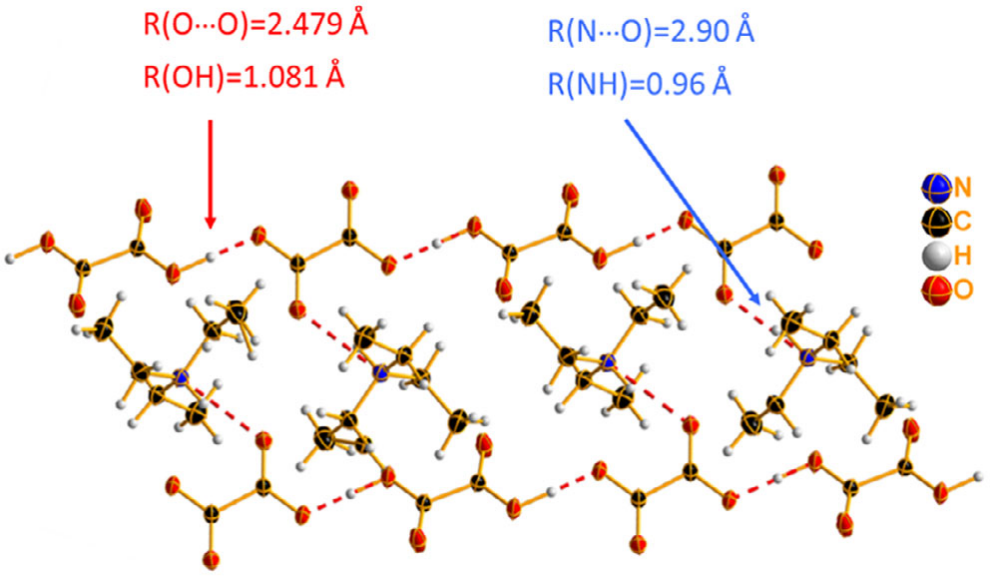
Crystal structure of triethylammonium hydrogen oxalate [TEA][OOCCOOH] showing H‐bonded wires alternately built by dicarboxylate acid molecules and doubly negative charged oxalate anions. Thereby, the two OH groups of the molecule serve as H‐bond donors to the neighbored oxygen acceptors of the oxalate anions. The H‐bonds characterized by anion–anion ^−^OH···OC^−^ interaction and flanked by cation‐anion ^+^NH···O^−^ interaction. The H‐bonds are denoted by dashed lines. In the ORTEP representation, the atoms are represented by ellipsoids at 50% probability levels.

**TABLE 1:** Hydrogen bond donor–acceptor distances (in Å) and angles in the crystalline structures of triethylammonium hydrogen oxalate and triethylammonium malonate.

	[TEA][OOCCOOH]	[TEA][OOC(CH_2_)COOH]
*R*(NH···O)/Å	1.877	1.875
*R*(OH···O)/Å	1.398	1.387
*R*(N···O)/Å	2.800	2.735
*R*(O···O)/Å	2.479	2.479
*R*(N─H)/Å	0.960	0.886
*R*(O─H)/Å	1.081	1.094
*R*(C─O)/Å^a^	1.246	1.242
*R*(C─O)/Å^b^	1.267	1.265
∠(O,H,O)	179.4	174.8
∠(N,H,O)	160.4	163.5

a CO bond involved in the moderately strong H‐bonds ^+^NH···OC^−^.

b CO bond involved in the significantly stronger H‐bonds ^−^OH···OC^−^.

Figure 2a shows the X‐ray structure of triethylammonium hydrogen malonate. We observe that the hydrogen malonate anions are arranged in infinite single‐H‐bonded wires. Herein, the OH group of each anion is oriented at a carboxylate O atom of the next anion in the wire. The H‐bonds ^−^OH···O^−^ between the anions are again flanked by H‐bonds ^+^NH···O^−^ between the triethylammonium cation and hydrogen malonate anion. These H‐bonds show typical H‐bond behavior, as known for molecular systems. The N···O distance is 2.735 Å and the NH···O distance is 1.875 Å. The H‐bond angle ∠(N,H,O) is 163.5°, also indicating that the H‐bonds are moderate despite the Coulomb attraction between the opposite charged ions. In stark contrast, the ^−^OH···O^−^ type H‐bonds between the anions in the anionic wires are very strong despite the repulsive Coulomb interaction. The O···O distance is only 2.479 Å and the OH···O distance is 1.388 Å. The H‐bond angle ∠(O,H,O) is 174.8°, thus close to being linear. Within this strong ^−^OH···O^−^ type H‐bonds, the OH bond length is *R*(OH) = 1.09 Å and thus elongated more than 10 pm compared to the typical gas‐phase value of monomeric carboxylic acids, being 0.972 Å in formic acid and 0.971 Å in acetic acid [37, 38, 39]. The typical OH bond lengths for water are 0.945 Å in the gas phase, 0.98 Å for liquid water, and 1.01 Å in ice [3]. Thus, the elongation of the OH bond between two anions exceeds the elongation of the OH bond in water and ice due to the H‐bond network. Such lengthening is inherent to strong H‐bonds and is a consequence of weakening of the covalent OH bond by the ^−^OH···O^−^ interaction. Further evidence for only moderate H‐bonds ^+^NH···O^−^ between cation and anion but strong ^−^OH···O^−^ between the anions is given by the CO bond lengths of the carboxylate group (see Figure 2b). The CO bond involved in the moderately strong H‐bonds ^+^NH···OC^−^ is 1.242 Å, whereas the CO bond involved in the significantly stronger H‐bonds ^−^OH···OC^−^ is elongated to 1.265 Å. This means that the CO bond involved in the anion–anion H‐bonds is 0.023 Å longer than the CO bond involved in the cation–anion H‐bonds, clearly indicating much stronger H‐bonds between two anions compared to those between cation and anion. Recently, Steiner and Saenger correlated the OH and H···O distance of 125 OH···O interactions found in neutron diffraction crystal structures extracted from the Cambridge Structural Database [39, 40]. This data included different kinds of H‐bonds: OH···O, OH···O^─^, ^─^OH···O, and ^−^OH···O^−^. Using their relation, our measured intermolecular H···O distance of about 1.388 Å predicts an OH bond length of about 1.1 Å, which is exactly the OH bond length we measured. Here, we conclude that the H‐bonds between like charged ions are surprisingly strong but do not differ from classical H‐bonds. Considering that the covalent OH bond distances are underestimated by about 0.1 Å in X‐ray crystallography, the proton can be assumed to be almost equally shared between the two O‐atoms, resulting in the extreme case of the centered H‐bonds O···H···O. This suggests that the PILs may be efficient proton‐conducting materials [41, 42].

**FIGURE 2:**
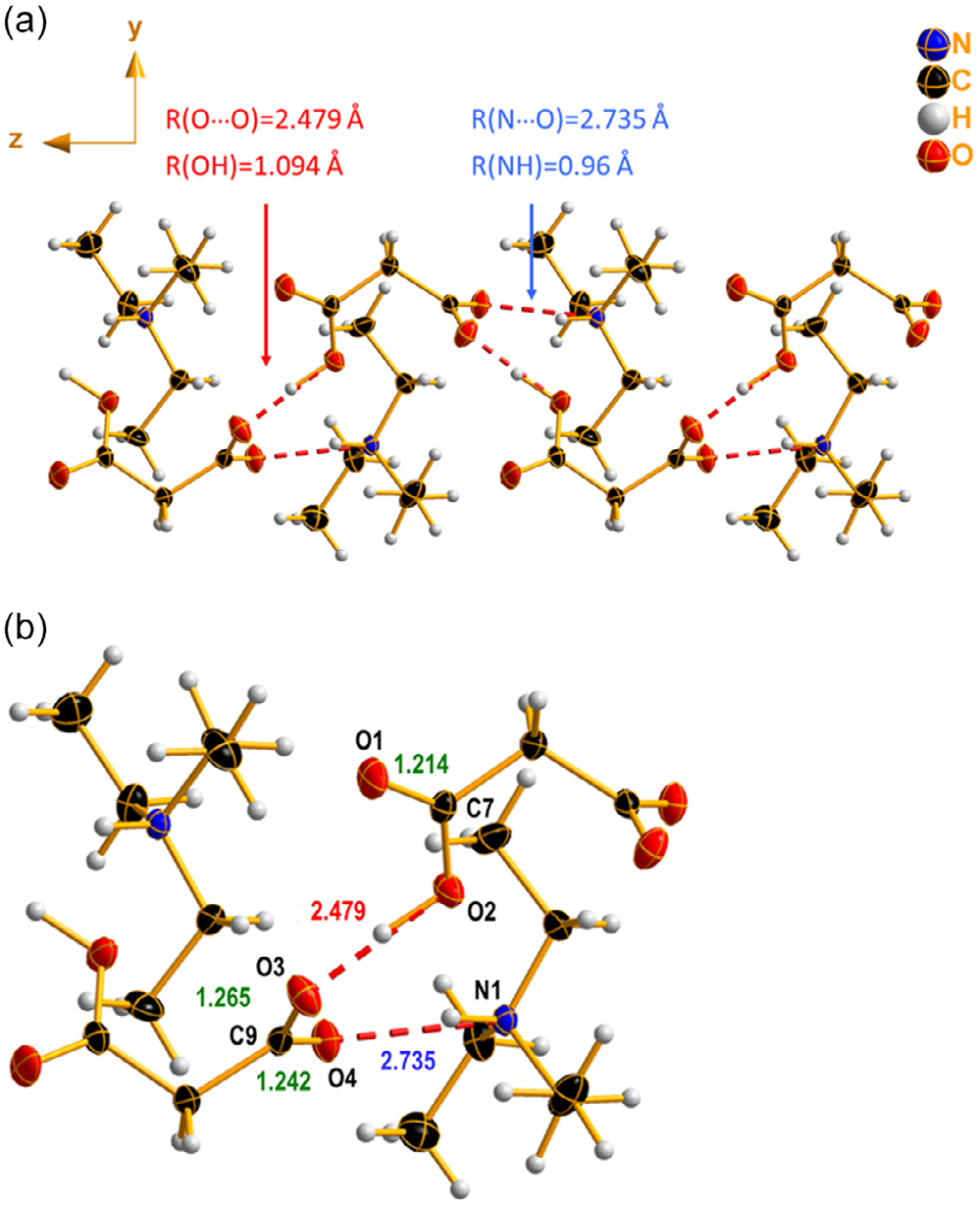
(a) Crystal structure of triethylammonium hydrogen malonate [TEA][OOC(CH_2_)COOH] showing anionic wires characterized by anion–anion ^−^OH···OC^−^ H‐bonds and flanked by cation–anion ^+^NH···O^−^ H‐bonds. The H‐bonds are denoted by dashed lines. In the ORTEP representation, the atoms are represented by ellipsoids at 50% probability levels. (b) Enlarged section of the X‐ray structure of triethyl ammonium hydrogen malonate showing the H‐bond distances *d*(O2H···O3) between anion and anion and the H‐bond distances *d*(N1···O4) between cation and anion. Additionally, the covalent CO bond distances *d*(C7‐O1) of the carbonyl group and *d*(C9‐O2) and *d*(C9‐O4) of the carboxylate group are shown. Distances are given in Å.

### 2.2 H‐Bonded Anionic Wires Preserved in the Liquid State Shown by NMR and IR Spectroscopy

Now we address the question, whether the anionic wires and the H‐bonds, ^−^OH···O^−^ and ^+^NH···O^−^, survive the melting from the solid into the liquid state of the triethylammonium hydrogen malonate. For finding structural evidence, we used NMR and IR spectroscopy. While ^1^H NMR is commonly used to probe interionic H‐bonds, it is often limited by proton exchange and line broadening due to chemical exchange [29]. However, for the viscous triethylammonium hydrogen malonate, the proton exchange is slowed down at 298 K, so that we observed two separate signals for the OH and the NH protons around 14 and 10 ppm, respectively. Strongly downfield‐shifted OH protons of the carboxy group ‐ typically ranging between 12–15 ppm ‐ indicate very strong H‐bonds, as shown for carboxylic acids [43]. In Figure 3, we show the ^1^H NMR chemical shift range from 7 to 16 ppm as a function of temperature. The full ^1^H and ^13^C spectra can be found in the Supporting Information. The recorded spectra at 298 and 303 K clearly show two signals, which are referred to the downfield chemical shifts of the OH protons (red area) and NH protons (blue area). Additional evidence for this assignment of the signals is provided by DFT calculations at the B3LYP‐D3/6‐31+G* level of theory on structures showing the H‐bond patterns as observed in the X‐ray structures [44, 45, 46, 47]. Calculated proton chemical shifts were derived against the reference tetramethylsilane (TMS), similar to the experiment, and reflect our assignment of the signals in the measured ^1^H NMR spectra (Figure 3). The fact that the signals of OH and NH protons of the solid‐like calculated clusters are found in the same ppm range as those of the measured liquid state structures suggests that the structural features observed in the X‐ray patterns are mainly preserved in the liquid state of the supercooled PILs. Figure 3 also shows that above 303 K, the two signals coalesce to one due to fast exchange of the NH and OH protons.

**FIGURE 3:**
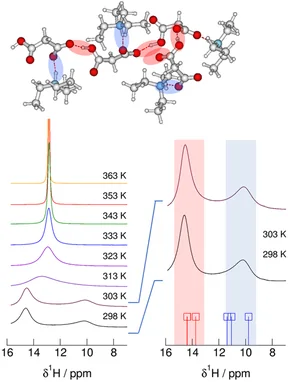
NMR proton chemical shifts *δ* ^1^H of the COOH and the NH groups observed for triethylammonium hydrogen malonate [TEA][OOC(CH_2_)COOH] as a function of temperature. At 298 K the proton exchange is obviously slow and we observe two broad signals at around 10 ppm and slightly above 14 ppm. They are assigned to chemical shifts *δ* ^1^H of the NH and the COOH protons, lying in the blue and red areas of the enlarged spectra. The assignment is supported by the DFT calculated proton chemical shifts (vs. TMS) of stable H‐bonds clusters [TEA^+^]_4_[^−^OOC(CH_2_)COOH]_4_ as indicated by the squares. At high temperatures the two signals coalesce due to fast proton exchange.

A valuable method for investigating the structural features present in the liquid triethylammonium hydrogen malonate is Fourier‐ transform infrared (FT‐IR) spectroscopy. We measured the IR absorption spectra in the liquid state for a broad range of temperatures between 298 and 353 K in 10 K steps. It has turned out that this PIL can be supercooled and measured below the melting point. Although covering wavenumbers between 900 and 4000 cm^−1^, we mainly focus on the CO stretching vibrational bands, which are usually observed between 1600 and 1800 cm^−1^. As discussed for the X‐ray structure of solid triethylammonium hydrogen malonate, the CO bond distances in the carboxy and carboxylate groups depend on the intermolecular interaction strength. Thus, the CO vibrational bands *ν*_CO_ are sensitive probes of H‐bonding, namely, in the manner of shifting to lower wavenumbers with increasing H‐bond strength. The X‐ray structure of the crystalline PIL showed that the two carboxylate oxygens are involved in strong H‐bonds ^−^OH···OC^−^ and moderate H‐bonds ^+^NH···OC^−^, as indicated by the differently elongated CO bond distances *R*(CO) = 1.265 Å and *R*(CO) = 1.242 Å (see Table 1). In contrast, the oxygen of the carboxyl group is not involved in H‐bonds, as expressed by the shortest CO bond distance *R*(CO) = 1.214 Å. The H‐bond dependence of the CO bond lengths should be reflected in the corresponding CO stretching frequencies *ν*_CO_. That is what we observe in the IR spectra shown in Figure 4 . We assign the CO stretching band with the highest frequency at 1720 cm^−1^ to the non‐H‐bonded CO in the carbonyl group. The redshifted CO stretches around 1600 cm^−1^ are referred to the CO bonds of the carboxylate group that are heavily involved in H‐bonds. This clear assignment is supported by DFT frequency calculations at the B3LYP‐D3/6‐31+G* level of theory on structures resembling the anionic wires observed in the X‐ray structures [44, 45, 46, 47]. In Figure 4, we show that the calculated frequencies of the non‐H‐bonded CO fall into the red area and the H‐bonded CO into the blue area in accordance with our assignment. Obviously, the structural H‐bond motifs observed in the crystal structure of hydrogen triethyl ammonium malonate are still present in the bulk liquid phase. The fact that the ^−^OH···OC^−^ interaction is stronger than the ^+^NH···OC^−^ interaction suggests that the interanion ^−^OH^…^O^−^ interactions are definitely based on H‐bonding between ions of like charge.

**FIGURE 4:**
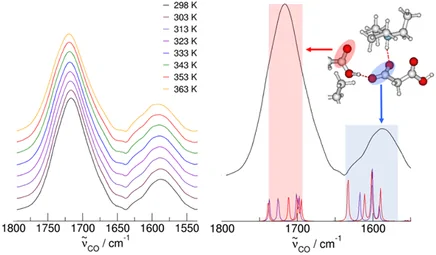
Attenuated total reflection (ATR) IR spectra of triethylammonium hydrogen malonate [TEA][OOC(CH_2_)COOH] showing two main vibrational bands in the CO stretching region. The vibrational band at around 1720 cm^−1^ is assigned to the nonbonded CO group of the COOH carboxyl group (red area), whereas the strongly redshifted bands at around 1580 cm^−1^ are assigned to H‐bonded CO in the carboxylate group (blue area), as shown in the structural motif and assigned by the arrows. The assignment is supported by the DFT calculated CO vibrational bands obtained from stable H‐bonds clusters [TEA^+^]_4_[^−^OOC(CH_2_)COOH]_4_.

### 2.3 DFT Calculated Neutral and Charged Clusters Mimic the Condensed Phase H‐Bond Motifs

Recently, this interpretation of interanion ^−^OH···O^−^ interactions as H‐bonds has been seriously challenged. Braga and coworkers argued that the ^−^OH···O^−^ interaction in potassium hydrogen oxalate cannot be regarded as a H‐bond despite the short O···O distance of 2.52 Å and the linearity of the geometry [20]. They questioned the anion–anion interaction from molecular electrostatic potential considerations and concluded that it is instead a “tugboat interaction,” controlling anion aggregation and minimizing anion–anion repulsions. They claimed that the anionic dimer would fall apart if the counter cations are removed. For demonstrating that we are dealing with real strong and directional H‐bonds within the anionic wires, we calculated neutral and charged stable H‐bond clusters [TEA^+^]_*m*_[^−^OOC(CH_2_)COOH]_4_ with *m* = 0–4 consisting of *m* triethylammonium cations and four hydrogen malonate anions as calculated at the B3LYP–D3/6–31+G* level of theory considering additional dispersion interaction as provided by Grimme's D3 method [44, 45, 46, 47]. In Figure 5, we show the neutral tetrameric cluster [TEA^+^]_4_[^−^OOC(CH_2_)COOH]_4_ followed by charged species, wherein successively triethylammonium cations are removed, resulting in overall charged species *Q* = 0, *Q* = 1−, *Q* = 2−, and *Q* = 3−. The clusters show the tetrameric anionic wires and the H‐bond patterns as observed in the X‐ray structures. The calculated binding energies Δ*E* per ion of the neutral and charged stable H‐bond clusters are −280.8 kJ mol^−1^ for *Q* = 0, −257.7 kJ mol^−1^ for *Q* = 1−, −201.8 kJ mol^−1^ for *Q* = 2−, and −73.5 kJ mol^−1^ for *Q* = 3−, as shown in Figure 6. Thus, even for the threefold negatively charged cluster [TEA^+^][^−^OOC(CH_2_)COOH]_4,_ the binding energies Δ*E* per ion are still negative. The overall binding energy for the *Q* = 3− cluster sums up to −367.5 kJ mol^−1^, which is similar to the calculated binding energy of the ion pair [TEA^+^][^−^OOC(CH_2_)COOH] as isolated from this structure, indicating attractive H‐bonding within the anionic wire despite the repulsive Coulomb interaction.

**FIGURE 5:**
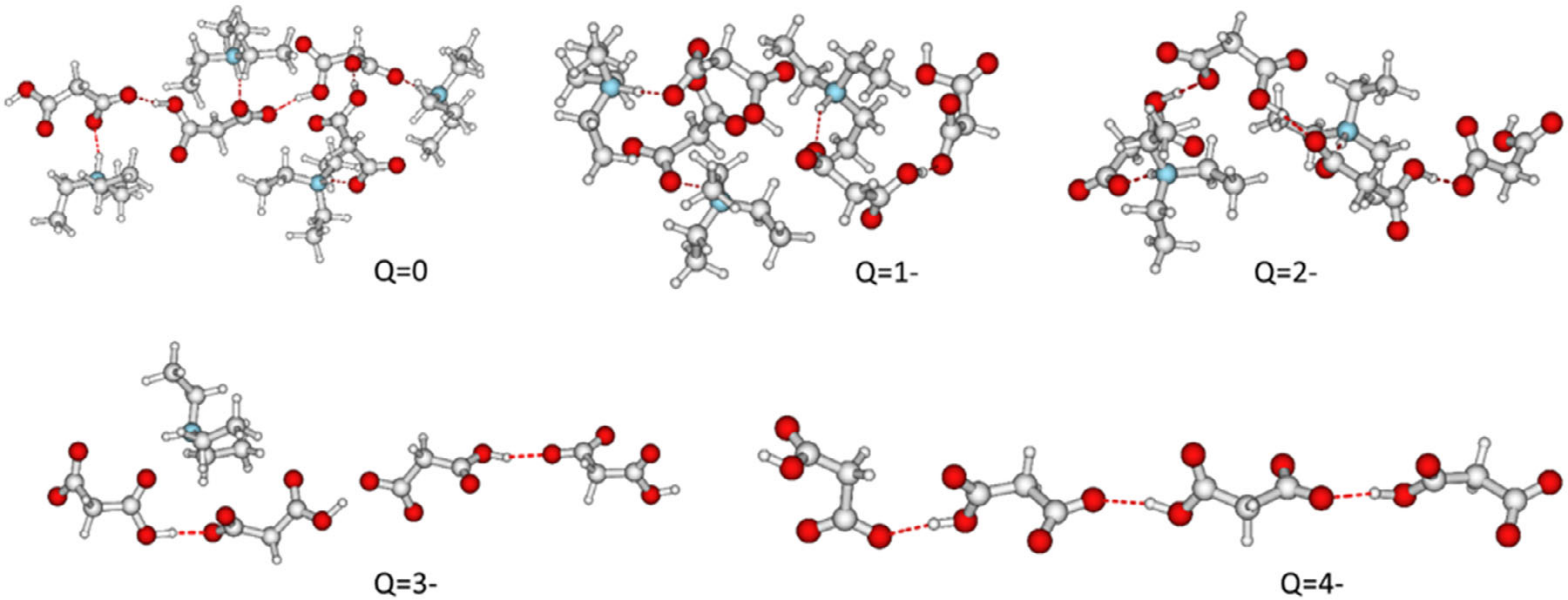
Neutral and charged stable H‐bonds clusters [TEA^+^]_*m*_[^−^OOC(CH_2_)COOH]_4_ with *m* = 0–4 consisting of *m* triethylammonium cations and four hydrogen malonate anions as calculated at the B3LYP–D3/6–31+G^*^ level of theory. The neutral tetrameric cluster [TEA^+^]_4_[^−^OOC(CH_2_)COOH]_4_ (*Q* = 0) is followed by charged species wherein successively triethylammonium cations are removed, resulting in charged species *Q* = 1−, *Q* = 2−, *Q* = 3−, and finally the pure linear anionic tetramer (*Q* = 4−).

**FIGURE 6:**
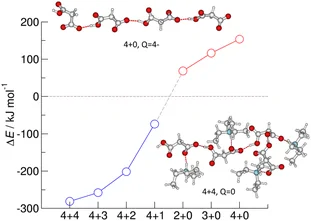
The calculated binding energies Δ*E* per ion of the neutral and charged stable H‐bonds clusters [TEA^+^]_*m*_[^−^OOC(CH_2_)COOH]_4_ with *m* = 0–4. The Δ*E* values are −280 kJ mol^−1^ for *Q* = 0, −250 kJ mol^−1^ for *Q* = 1−, −200 kJ mol^−1^ for *Q* = 2−, and −75 kJ mol^−1^ for *Q* = 3−. Thus, even for the threefold negatively charged cluster the binding energies Δ*E* per ion is still negative. The overall binding energy for the *Q* = 3− cluster sums up to −367.5 kJ mol^−1^, which is even higher than the calculated binding energy of an ion pair [TEA^+^][^−^OOC(CH_2_)COOH] in indicating attractive H‐bonding within the anionic wire despite the repulsive Coulomb interaction.

### 2.4 Double‐Charged Hydrogen Malonate Dimer Shows Robust Kinetic Stability

In Figure 6, we also show the energies per ion for the pure anionic clusters [HOOC(CH_2_)COO^−^]_*m*_ for *m* = 2,3,4. The positive Δ*E* values show that the repulsive Coulomb are stronger than the attractive H‐bonds between the anions. However, none of the anionic clusters falls apart during the geometry optimization and showed substantial kinetic stability, as observed for hydroxy‐ and carboxy‐functionalized cations of pyridinium‐based ILs [11, 12, 13, 14, 15, 16, 17]. This is demonstrated for the double‐charged hydrogen malonate dimer [HOOC(CH_2_)COO^−^]_2_ in more detail. The potential energy curve for the hydrogen‐bonded anionic dimer [HOOC(CH_2_)COO^−^]_2_, as calculated at the B3LYP–D3/6–31+G* level of theory, shows a minimum with a clear dissociation barrier preventing “Coulomb explosion” into separated anions. We achieved robust kinetic stabilities while dissociating the anionic dimer along the *R*(COH···OC) H‐bond stretching coordinate with respect to the energy of the isolated cations at Δ*E* = 0. For each intermolecular distance *R*(COH^…^OC), all other geometrical variables were optimized at each 0.5 pm step of the scan. The relaxed potential energy curve for the hydrogen malonate dimer [HOOC(CH_2_)COO^−^]_2_ is shown in Figure 7. We indicate the minimum energy Δ*E* = 123.89 kJ mol^−1^ at the equilibrium H‐bond distance *R*(H^…^O) = 1.701 Å. The distance *R*(O^…^O) = 2.688 Å is extremely short too despite the strong repulsive Coulomb interaction present due to only one methylene group spacer between the carboxyl and the carboxylate groups. The intermolecular oxygen–oxygen distance in this anionic dimer is even shorter than that in ice (2.72 Å), exhibiting strong hydrogen bonds and even 0.19 Å shorter than that of water dimers calculated at the same level of theory [40]. For the anionic hydrogen malonate dimer [HOOC(CH_2_)COO^−^]_2,_ we could calculate energy potential curves well beyond the activation barrier height. In Figure 7 we show the effective well depths ΔE* with respect to the transition state at ΔR*(H^…^O) that signals descent towards separated cations. For this anionic dimer, a distance of about 3.1 Å corresponds to an activation barrier height of about ΔE* = 18.3 kJ mol^−1^, showing the enormous strength of the H‐bonds.

**FIGURE 7:**
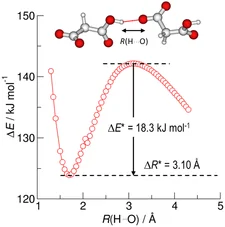
Potential energy curve for the hydrogen‐bonded anionic dimer [^−^OOC(CH_2_)COOH]_2_, as calculated at the B3LYP–D3/6–31+G^*^ level of theory, relaxing the structure for each hydrogen bond length *R*(COH···OC). The curve progression is characterized by a clear minimum energy Δ*E* of about 123.99 kJ mol^−1^ at the equilibrium bond distance *R*(COH···OC) = 1.70 Å. The dissociative energy profile shows a pronounced binding well with respect to the dissociated anions and effective equilibrium well depths ΔE* = 18.3 kJ mol^−1^ at ΔR* = 3.10 Å with respect to the transition state.

### 2.5 Natural bond orbital (NBO) Descriptors Strongly Correlate With NMR Proton Chemical Shifts and Vibrational Frequencies

The kinetically stable anionic dimers, trimers, and tetramers show that the short‐range donor–acceptor covalency forces successfully compete with the powerful long‐range electrostatic repulsions [15, 16, 17]. The characteristic covalency features of the H‐bonds ^−^OH···O^−^ can be readily recognized in the framework of the NBO analysis as distinctive *n*_O_ → σ*_OH_ donor–acceptor interactions expressed by the second‐order stabilization energies Δ*E* ^(2)^_*n *→ σ*_ (see Supporting Information) [48, 49, 50]. The charge from the oxygen lone‐pair orbitals of one of the carboxylate groups CO of a malonate anion is donated into the OH antibonding orbital of the carboxyl group of the next anion and so on. This way, the short‐range donor–acceptor covalency forces overcome the strong long‐range electrostatic repulsive forces, as expected for ions of like charge. Here, we show that the NBO approach is also suitable for describing the energetic and spectroscopic features observed for the hydrogen‐bonded anionic wires in hydrogen malonate.

In Figure 8, we show the NBO energy versus the spectroscopic descriptors NMR proton chemical shifts *δ* ^1^H and infrared vibrational frequencies *ν*_CO_ for the anionic dimers, trimers, and tetramers. As shown, the second‐order stabilization energies Δ*E* ^(2)^_*n *→ σ*_ are almost linearly correlated with the spectral descriptors calculated for the anionic clusters. The increasing Δ*E* ^(2)^_*n *→ σ*_ values are reflected by downfield chemical shifts *δ* ^1^H of about 2 ppm and redshifted vibrational frequencies *ν*_CO_ of about 43 cm^−1^. Despite the increasingly positive values for Δ*E* with increasing cluster size due to the strong repulsive Coulomb forces between the anions, the spectral signatures show typical behavior of enhanced H‐bonding ranging from dimer to trimer to tetramer. Obviously, the second‐order stabilization energies Δ*E* ^(2)^_*n *→ σ*_ seems to be a suitable measure for the strength of H‐bonding. We demonstrate the usefulness of NBO analysis and highlight that NMR and IR properties are sensitive probes of hydrogen bonding and suitable for characterizing the interaction strengths between ions of like charge. Overall, the NBO parameters also indicate the covalent nature of hydrogen bonding in anionic wires.

**FIGURE 8:**
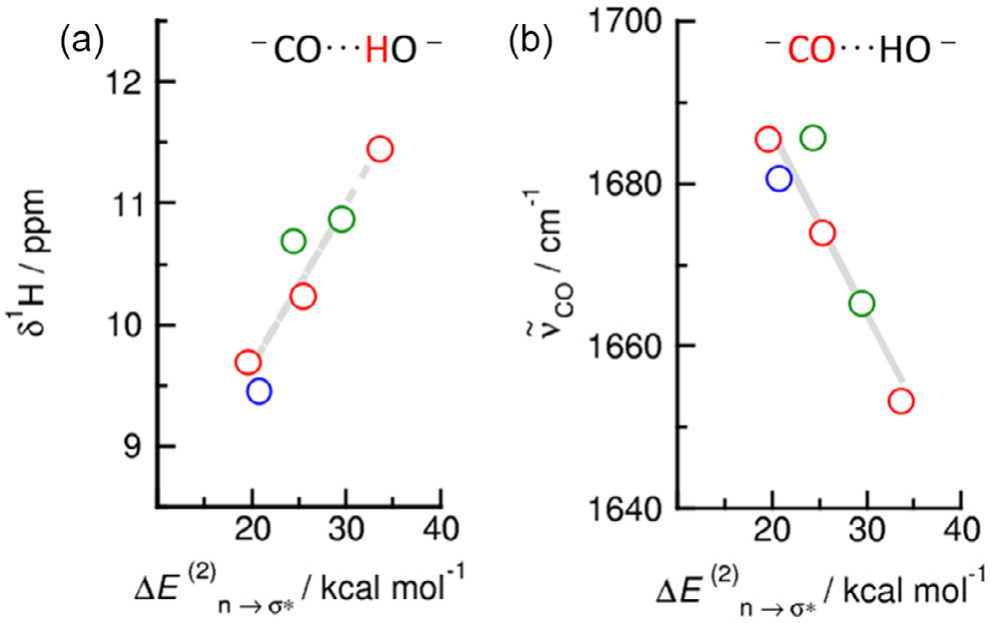
NMR proton chemical shifts, *δ* ^1^H (relative to the standard tetramethylsilane, TMS) (a) and IR vibrational frequencies, *ν*_CO_ (b) of hydrogen malonate dimers (blue), trimers (green) and tetramers (red) plotted versus NBO‐calculated second‐order stabilization energies Δ*E* ^(2)^_*n *→ σ*_. The almost linear dependences indicate the strong relation between NBO stabilization energies with geometric and spectroscopic properties of the anionic clusters.

Our combined experimental and theoretical approach shed some light into the vivid debate about so‐called “AEHB” [23, 24, 25, 26, 27]. This study suggests that the term IAHB is more suitable to describe the strong noncovalent interaction between ions of like charge [28, 29]. Nevertheless, our findings also challenge traditional electrostatic expectations and emphasize the important role of charge transfer and polarization for interionic H‐bonding, as expressed by spectral NMR and IR signatures. We found convincing structural and spectroscopic evidence that strong IAHB can even overcome the electrostatic repulsion. Thus, the claim of AEHB at least inspired the discussion about the nature of H‐bonding between ion of like charge and provoked experimental evidence for this kind of H‐bonds.

## 3 Conclusion

To summarize, we have studied the synthesis and solid‐state structural properties of triethylammonium hydrogen oxalate [TEA][OOCCOOH] and triethylammonium hydrogen malonate [TEA][OOC(CH_2_)COOH] PILs. X‐ray crystallography showed that the OH proton of the carboxy group of the anion forms single H‐bonds to one of the accepting oxygens of the carboxylate group of another anion ^−^COH···OC^−^. The NH proton of the triethylammonium cation forms single H‐bonds to the other accepting oxygen of carboxylate group of each anion ^+^NH···O^−^ type H‐bonds. Surprisingly, the H‐bonds ^−^OH···O^−^ between the anions are stronger than the H‐bonds ^+^NH···O^−^ between cation and anion, finally resulting in the formation of anionic wires despite the repulsive Coulomb interaction between the ions of like charge. ^1^H NMR and ATR IR spectroscopy provided strong evidence that the structural motifs observed in the solid, including the anionic wires, are still present in the bulk liquid phase. DFT‐calculated charged clusters up to *Q* = 4− exhibit appreciable kinetic stability and show characteristic structural and spectroscopic signatures of H‐bonds, despite the powerful Coulomb repulsion. Overall, our integrated experimental and computational investigation provides compelling evidence for the formation of anionic wires, challenging the traditional electrostatic expectations. The highly delocalized protons in the wires hold promise for materials with excellent proton conductivity. This strategy for probing interionic H‐bonding can be extended to a wide range of ionic environments such as promising “water‐in‐salt” and “solvate‐ionic‐liquids” electrolytes [51, 52, 53, 54, 55].

## Author Contributions

**Maham Azhar**: investigation (lead), data curation (equal), formal analysis (lead), writing – original draft (equal), writing – review and editing (equal). **Lasse Hunger**: data curation (equal), formal analysis (supporting). **Loai Al‐Sheakh**: data curation (supporting), formal analysis (supporting). **Andreas Appelhagen**: formal analysis (supporting). **Lennart Kruse**: formal analysis (supporting), writing – review and editing (equal). **Ralf Ludwig**: conceptualization (lead), project administration (lead), funding acquisition (lead), writing – original draft (equal), writing – review and editing (equal), resources (lead).

## Funding

This study was supported by the Deutsche Forschungsgemeinschaft (SPECTRE Research Training Group (507189291/GRK 2943), 470038970/LU‐506/17‐1, 517661181/LU‐506/18‐1).

## Conflicts of Interest

The authors declare no conflicts of interest.

## Supporting information

A detailed synthesis procedure, corresponding ^1^H and ^13^C NMR spectra, experimental details of infrared and X‐ray spectroscopic measurements, as well as information on the theoretical calculations are available in the Supporting Material. The authors have cited additional references within the Supporting Information [56, 57].

## Data Availability

The data that support the findings of this study are available in the Supporting Information of this article and from the corresponding author upon reasonable request.
