# Supplementary material for: Structural and Spectroscopic Evidence of Hydrogen Bonded Anionic Wires in Protic Ionic Liquids
Source: Chemphyschem. 2026 Sep 29;27(19):e70581. doi: 10.1002/cphc.70581 (PMC13624368; doi:10.1002/cphc.70581)
Supplement: Supplementary file 1 [file CPHC-27-e70581-s001.pdf]

Electronic Supporting Information (ESI)

Structural and Spectroscopic Evidence of Hydrogen Bonded Anionic Wires in Protic Ionic Liquids

Maham Azhar[a], Lasse Hunger[a], Loai Al-Sheakh[a], Andreas Appelhagen[a], Lennart Kruse[a], Ralf Ludwig[a,b,c]*

[a] Universität Rostock, Institut für Chemie, Abteilung für Physikalische Chemie, Albert-Einstein-Str. 27, 18059 Rostock, Germany; E-mail: ralf.ludwig@uni-rostock.de

[b] Department LL&M, University of Rostock, Albert-Einstein-Str. 25, 18059 Rostock, Germany

[c] Leibniz-Institut für Katalyse an der Universität Rostock e.V. Albert-Einstein-Str. 29a, 18059 Rostock, Germany

Table of contents

S1. Synthesis 2
S2. NMR Spectra 2
S3. IR 6
S4. X-Ray 6
S5. Gaussian 7

S1. Synthesis

The starting point of our investigation has been the synthesis of the ionic liquids or low melting salts using dicarboxylic acids together with the triethylamine. As educts, we used triethylamine (TEA) and two alkanedicarboxylic acids ($HOOC(CH_2)_nCOOH$) with n = 0,1, namely ethanedionic (oxalic) and propanedionic (malonic) acids for studying the effect of adding one methylene groups CH_2 between the two carboxylic groups. For general synthesis round bottom flask with a magnetic stirrer was charged with the acid in the solid powdered form. Under the cooling bath, equimolar amount of triethylamine was added slowly through dropping funnel in to flask. Then, let it stir for few hours. Depending on the dicarboxylic acid used, the resulting mixture was either ionic liquid or low melting salts.

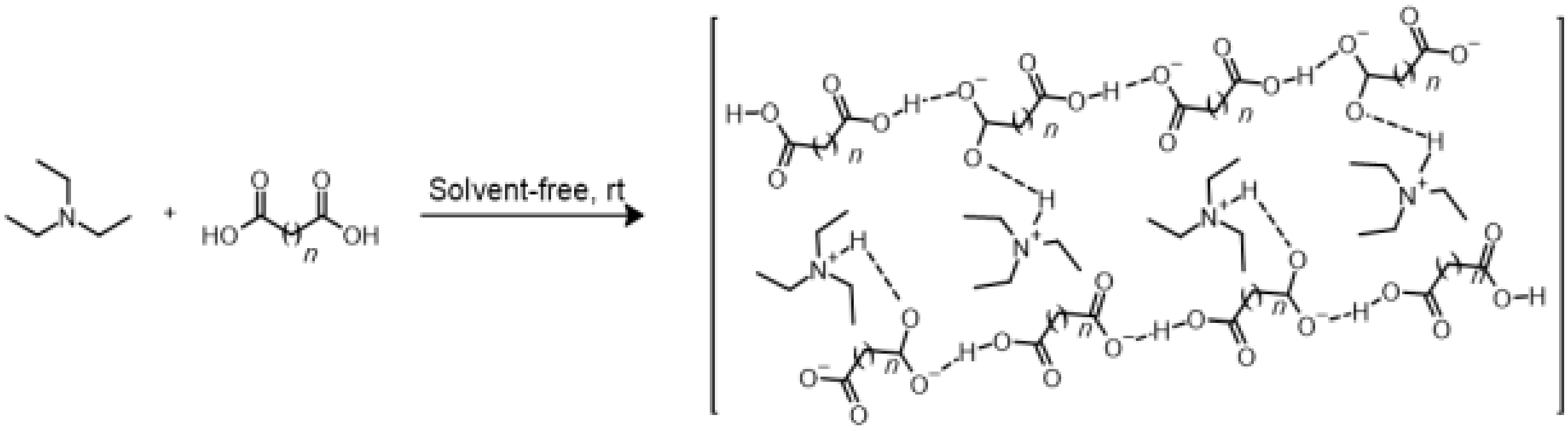

Scheme S1. Reaction scheme for the synthesis of triethylammonium hydrogen dicarboxylate protic ionic liquids [TEA][$OOC(CH_2)_nCOOH$] with n=0,1 obtained from triethyl amine (TEA) and ethanedioic acid HOOCCOOH (n=0) and propanedioic acid $HOOC(CH_2)COOH$ (n=1), respectively.

S2. NMR Spectra

Purity and success of the synthesis have been checked via ^1H and ^{13}C NMR spectroscopy.

[TEA][OOCCOOH]

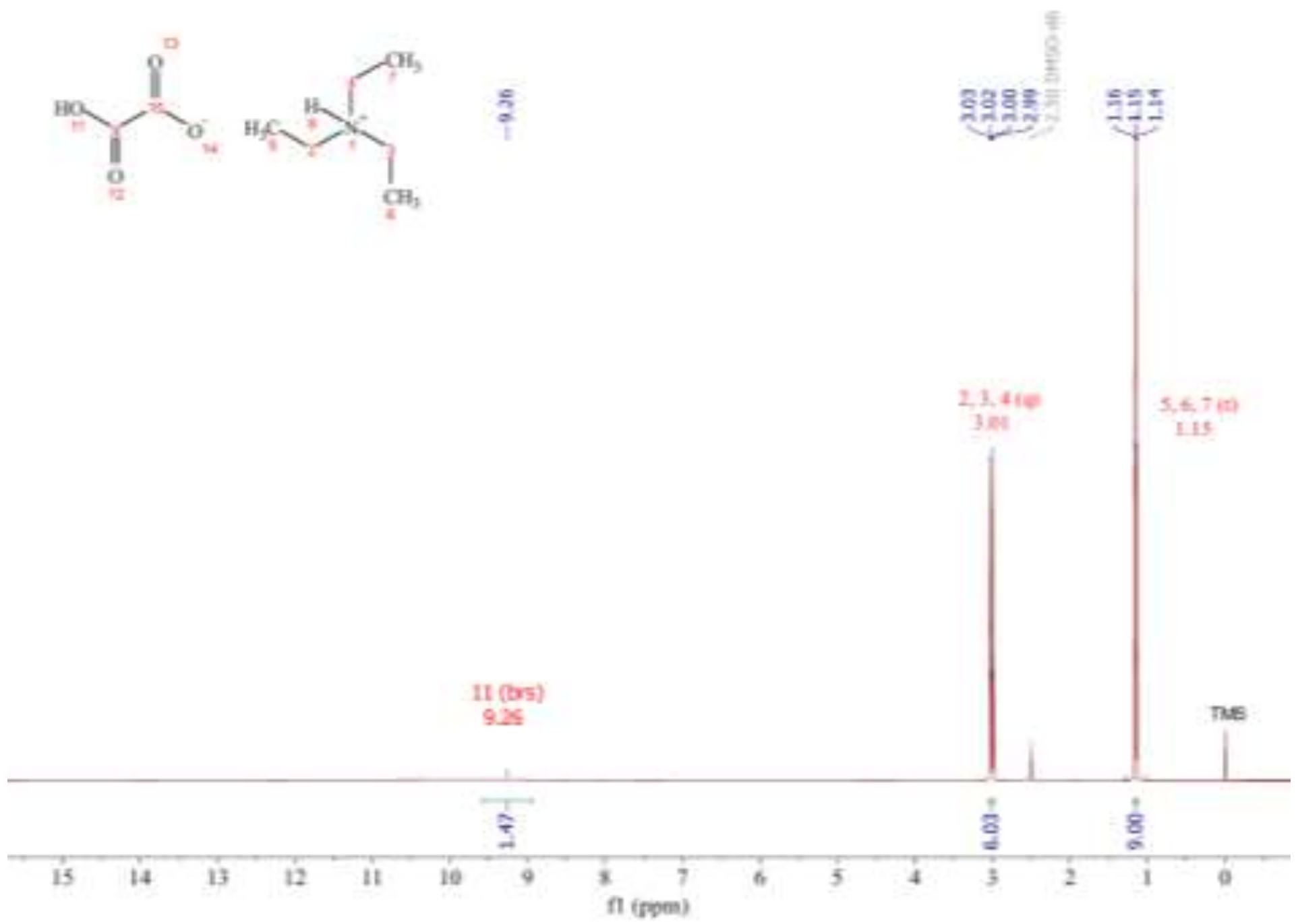

Figure S1. ^{1}H NMR spectrum of [TEA][OOCCOOH] in DMSO-d_6.

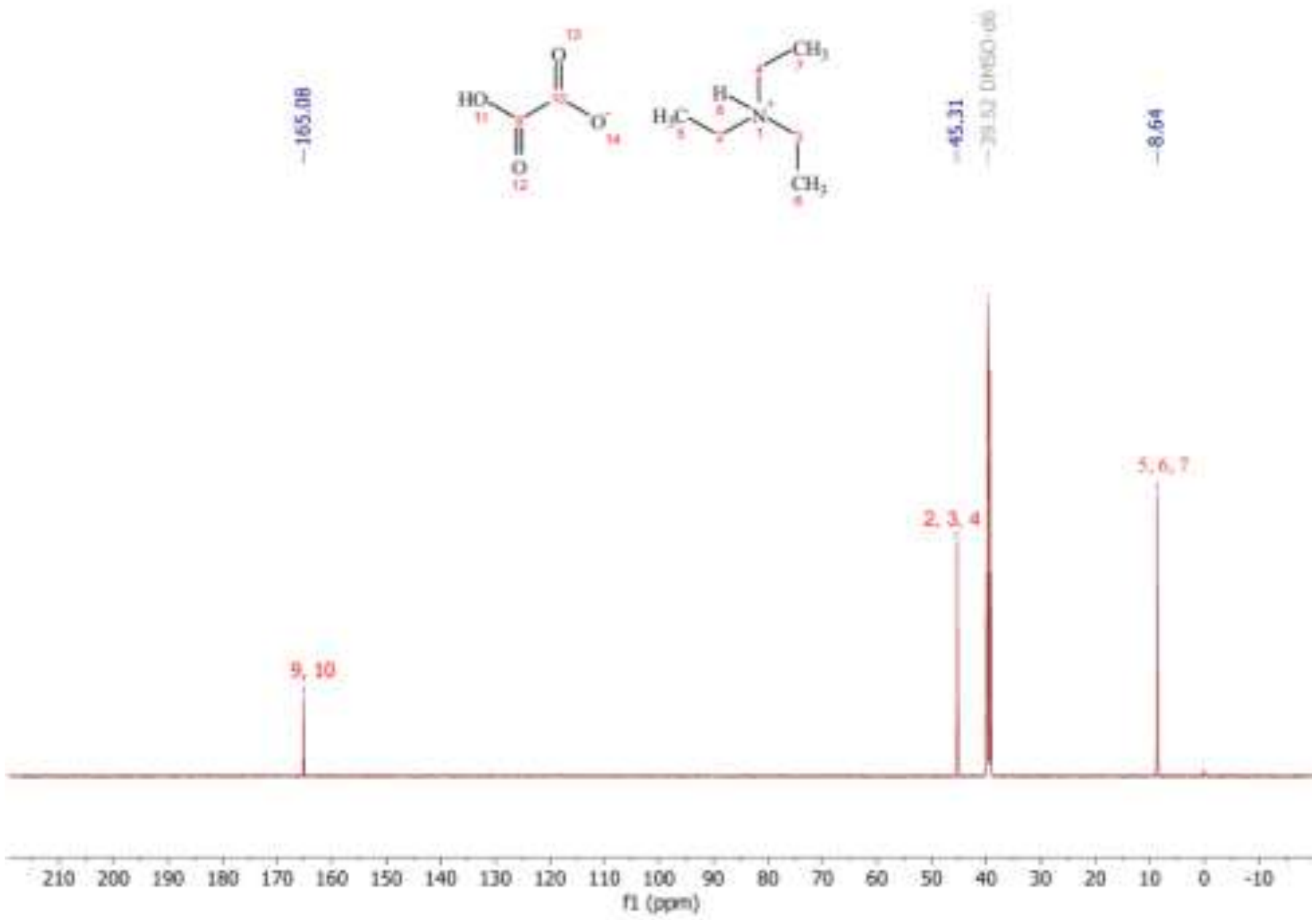

Figure S2. ^{13}C NMR spectrum of [TEA][OOCCOOH] in DMSO-d_6.

^{1}H NMR (500MHz, DMSO-d_6): δ 9.26 (br s, 2H), 3.01 (q, J = 7.2 Hz, 6H), 1.15 (t, J = 7.25 Hz, 9H).

^{13}C{1H} NMR (125 MHz, DMSO-d_6): δ 165.08, 45.31, 8.64.

[TEA][OOC(CH$_2$)COOH]

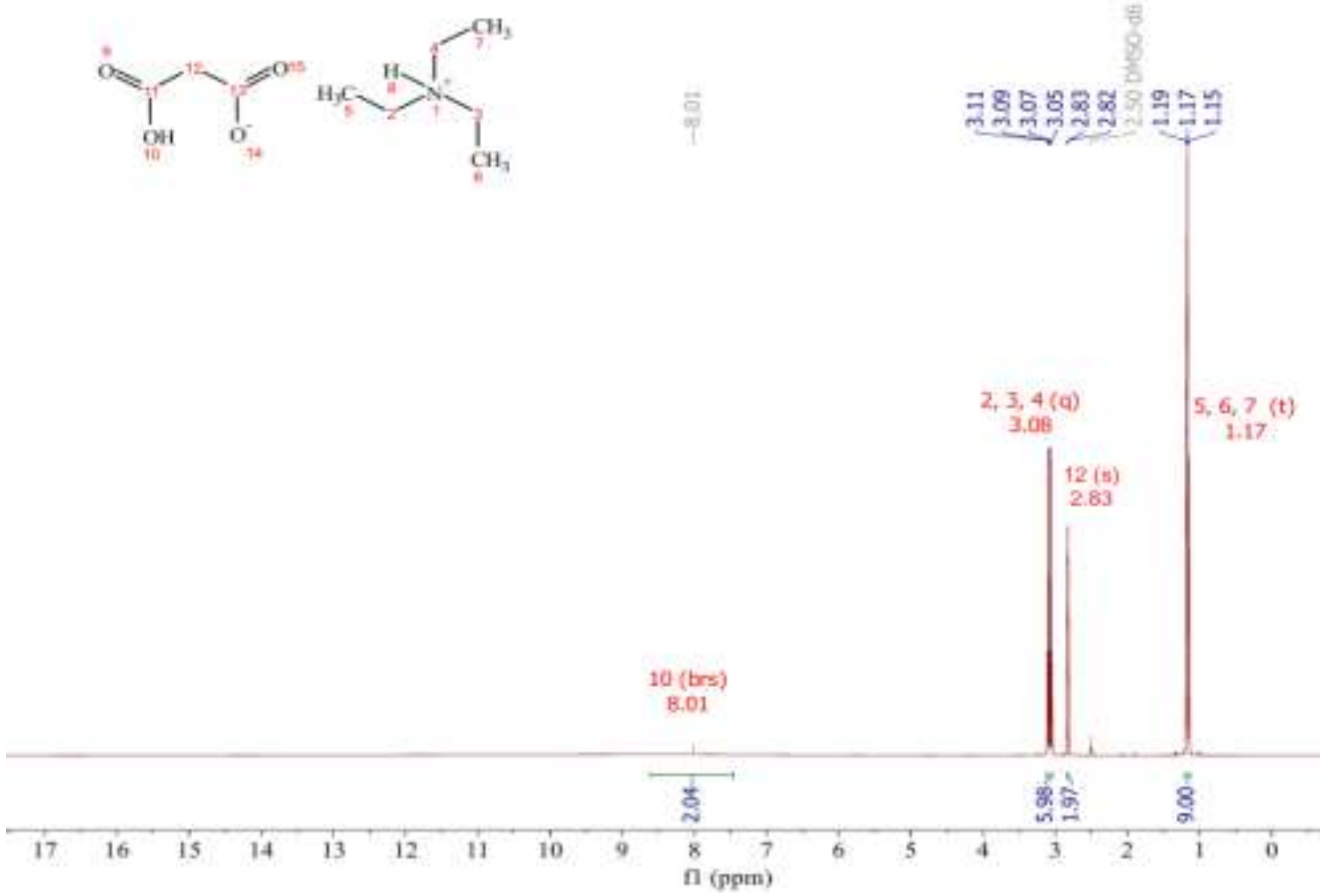

Figure S3. ^{1}H NMR spectrum of [TEA][OOC(CH$_2$)COOH] in DMSO-d_6.

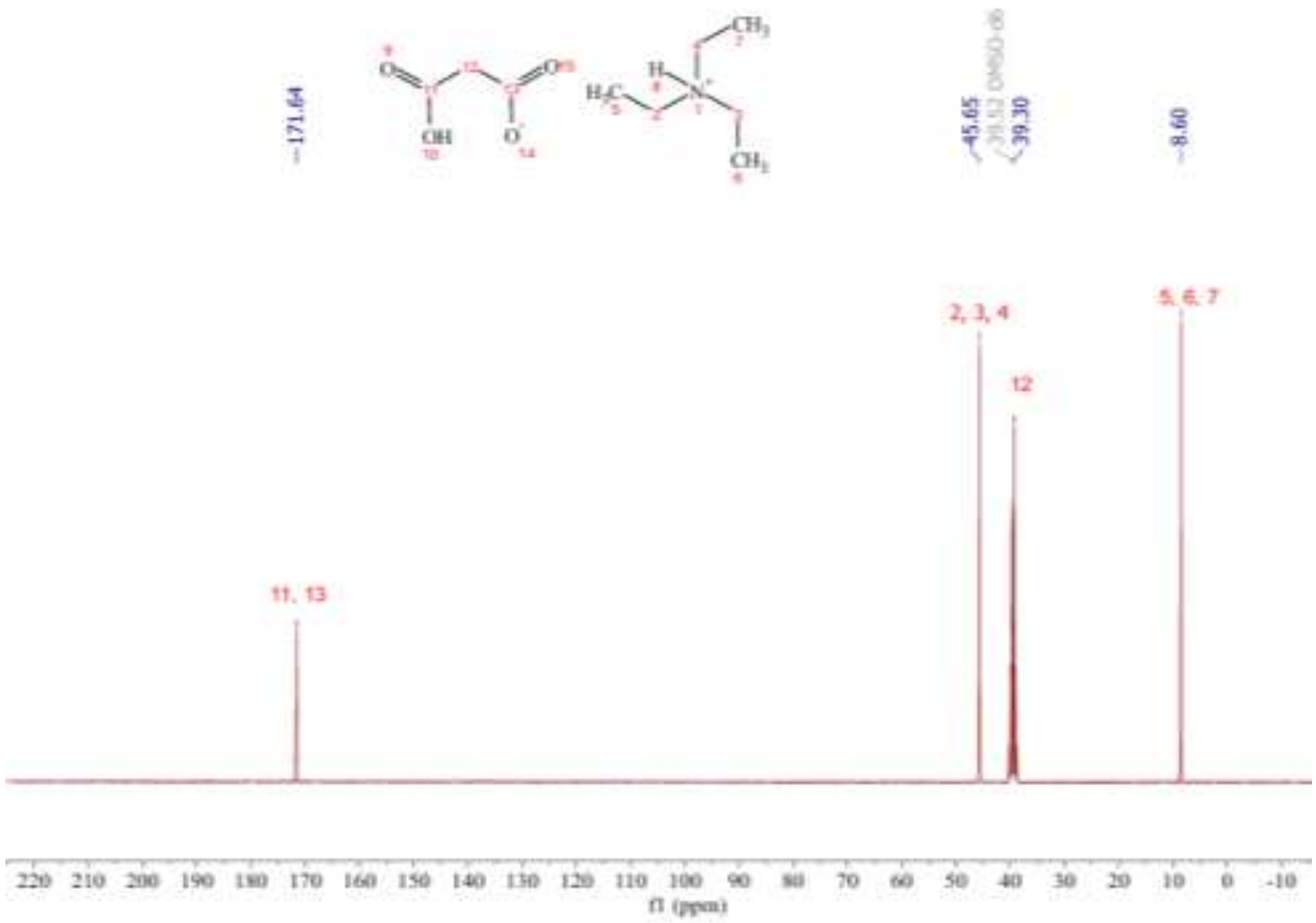

Figure S4. ^{13}C NMR spectrum of [TEA][OOC(CH$_2$)COOH] in DMSO-d_6.

^{1}H NMR (400MHz, DMSO-d_6): δ 8.01 (br s, 2H), 3.08 (q, J = 7.3 Hz, 6H), 2.83 (s, 2H), 1.17 (t, J = 7.3 Hz, 9H).

^{13}C{1H} NMR (101 MHz, DMSO-d_6): δ 171.64, 45.65, 39.30, 8.60.

For temperature-dependent NMR measurements the ionic liquid [TEA][$CH_2(COO)_2H$] was dried in high vacuum ($p < 10^{-5}$ mbar), transferred into standard 4 mm NMR tubes and flame-sealed in vacuo. The 4 mm tube was then inserted in a standard 5 mm outer tube and DMSO with TMS (1% v/v) is added for calibration. Spectra were recorded on a Bruker Avance Neo 500 MHz at 298 K and for a temperate range between 303 and 363 K in 10 K steps.

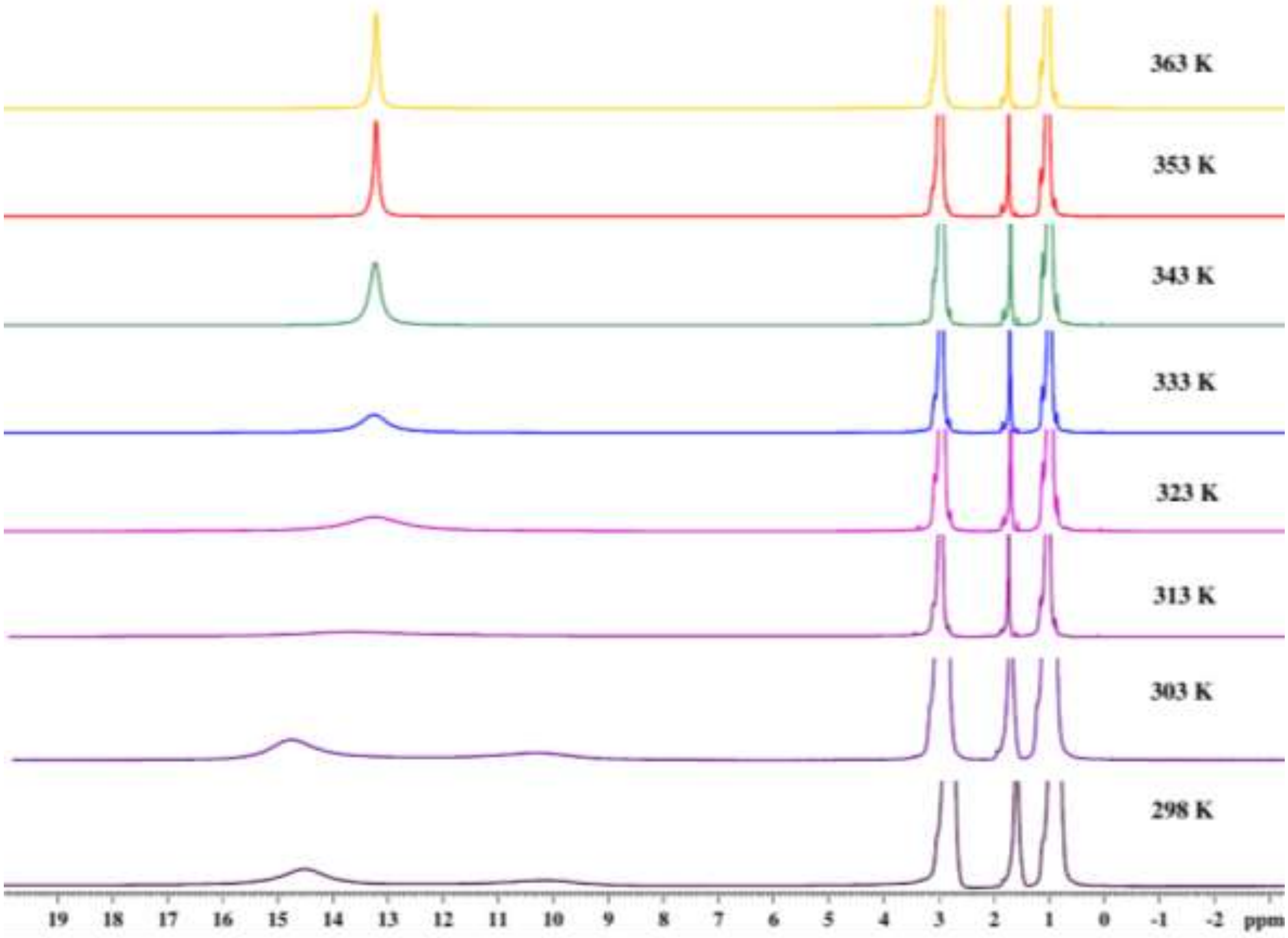

Figure S5. Temperature-dependent ^{1}H NMR spectra of [TEA][$OOC(CH_2)COOH$] between 298 K and 363 K.

S3. IR

Attenuated total reflection (ATR) mid-infrared (MIR) measurements of the IL [TEA][OOC(CH_2)COOH] were performed on a Bruker Vertex 70 FTIR spectrometer equipped with a Globar, KBr beamsplitter and a GladiATR™ system from PIKE Technologies. Heating of the ATR plate and the diamond crystal was realized by the built-in GladiATR™ temperature control with an accuracy of ± 0.5°C.

Spectra were recorded with 128 scans per spectrum at a spectral resolution of 1 cm^{-1} using the OPUS 7.5 spectroscopy software from BRUKER. The aperture setting was 4 mm and the scanner velocity was 7.5 kHz. The Power Spectrum phase correction and Blackman-Harris 3-Term apodization function were employed. Shown spectra were processed in terms of atmospheric compensations and baseline corrections using the OPUS 7.5 software.

S4. X-Ray

The crystal structure of triethylammonium hydrogen oxalate and of triethylammonium hydrogen malonate have been determined by X-ray crystallography with approximate size and quality was suited for structure analysis by X-ray. The X-ray quality crystal was selected in Fomblin YR-1800 perfluoroether at ambient temperature. The sample was cooled to 123(3) K during measurement. The data were collected on a Bruker KAPPA APEX II diffractometer using Mo Kα radiation (λ = 0.71073). The structures were solved by direct methods (*SHELXS-2013*) and refined by full-matrix least squares procedures (*SHELXL-2013*).[32,33] Semi-empirical absorption corrections were applied (*SADABS*).[34] All non-hydrogen atoms were refined anisotropically, hydrogen atoms were included in the refinement at calculated positions using a riding model.

The crystal structures of triethylammonium hydrogen oxalate and of triethylammonium hydrogen malonate can be found in the CSD under

https://www.ccdc.cam.ac.uk/structures/Search?Ccdcid=2386822&DatabaseToSearch=Published

https://www.ccdc.cam.ac.uk/structures/Search?Ccdcid=2386827&DatabaseToSearch=Published

S5. Gaussian

Additional evidence for the assignment of the IR and NMR signals is provided by DFT calculations on structures showing the H-bonds patterns as observed in the X-ray structures.[44-47]

We calculated neutral and charged H-bonded clusters $[TEA^+]_m[HOOC(CH_2)COO^-]_4$ with m=1-4 consisting of m triethylammonium cations and four hydrogen malonate anions at the B3LYP–D3/6–31+G* level of theory considering dispersion interaction as provided by Grimme's D3 method.[44-47] Additionally, we optimized the pure anionic clusters $[HOOC(CH_2)COO^-]_k$ with k=1-4. We point out that the counterpoise (CP) correction of Boys and Bernardi has been questioned recently [56, 57]. Intrinsic CP artifacts for ion pair clusters add to fundamental concerns that the CP procedure is generally unreliable for correcting basis superposition error [58]. Thus, a CP correction is not applied in this work. For all optimized neutral and charged clusters we calculated the vibrational frequencies and the ^{1}H chemical shifts (vs TMS) for comparison with IR and NMR experiments. Furthermore, we utilized natural bond orbital (NBO) analysis to study the donor-acceptor interaction strengths and the covalency features of the hydrogen bonds.[48-50] Here, the interaction strength is expressed by the second-order stabilization energy $\Delta E^{(2)}_{n\rightarrow\sigma^*}$. The charge from an oxygen lone pair orbital of one of the carboxylate groups is donated into the OH antibonding orbital of the carboxyl group of the next anion and so on. In Fig. S6 a corresponding 2D and 2D contour plot is shown, which highlight the orbital overlap.

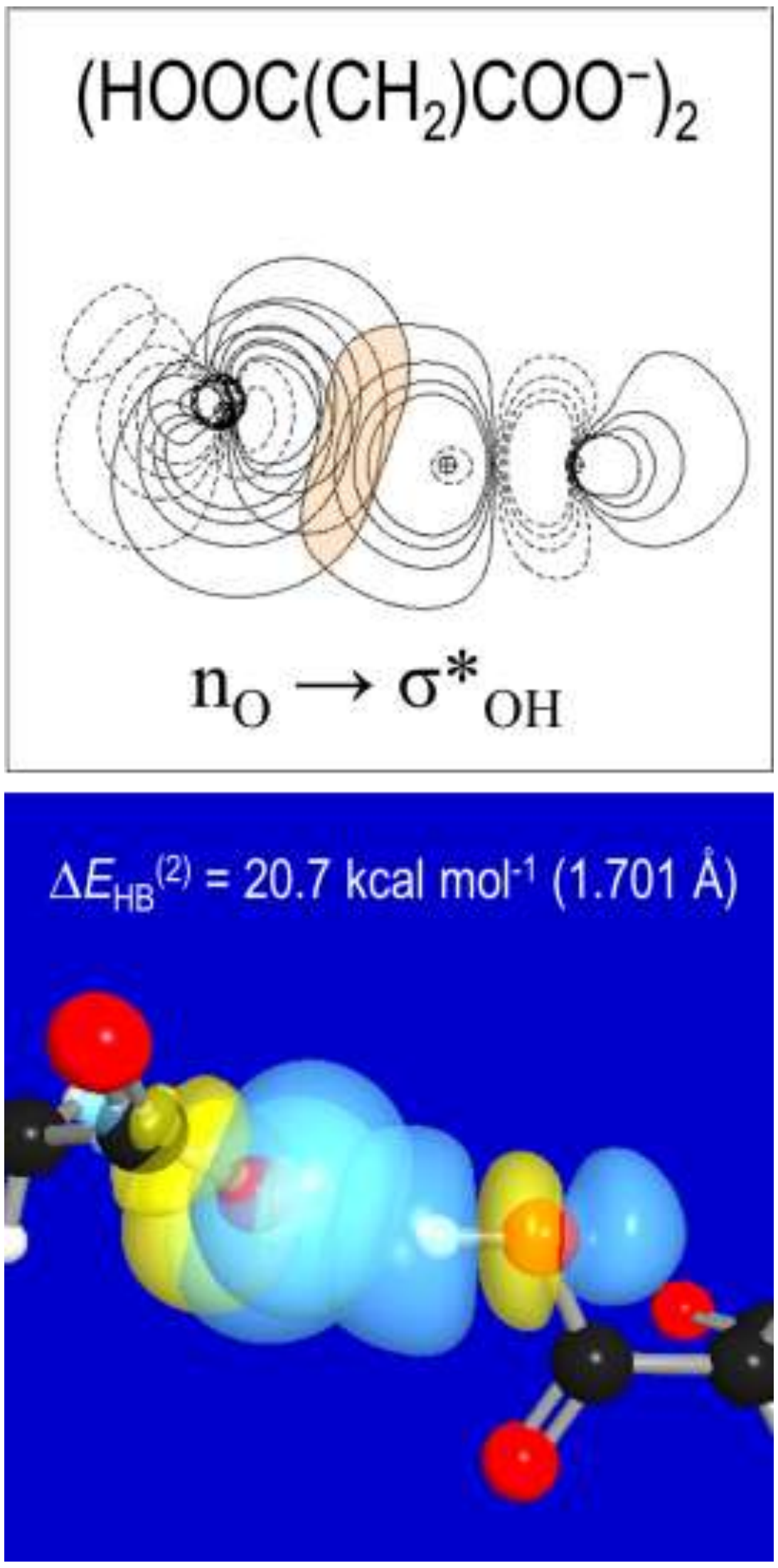

Figure S6. NBO 2D contour and 3D overlap diagrams for $n_O \rightarrow \sigma^*_{OH}$ donor–acceptor interaction in the anionic dimer $(HOOC(CH_2)COO^-)_2$ equilibrium geometry, with estimated second-order $\Delta E^{(2)}_{n\rightarrow\sigma^*}$ = 20.7 kcal·mol^{-1} interaction.
